# Fear conditioning of SCR but not the startle reflex requires conscious discrimination of threat and safety

**DOI:** 10.3389/fnbeh.2014.00032

**Published:** 2014-02-28

**Authors:** Dieuwke Sevenster, Tom Beckers, Merel Kindt

**Affiliations:** ^1^Department of Clinical Psychology, Faculty of Social and Behavioural Sciences, University of AmsterdamAmsterdam, Netherlands; ^2^Amsterdam Brain and Cognition, University of AmsterdamAmsterdam, Netherlands; ^3^Faculty of Psychology and Educational Sciences, Department of Psychology, University of LeuvenLeuven, Belgium

**Keywords:** fear conditioning, awareness, skin conductance response, fear potentiated startle, contingency learning

## Abstract

There is conflicting evidence as to whether awareness is required for conditioning of the skin conductance response (SCR). Recently, Schultz and Helmstetter (2010) reported SCR conditioning in contingency unaware participants by using difficult to discriminate stimuli. These findings are in stark contrast with other observations in human fear conditioning research, showing that SCR predominantly reflects contingency learning. Therefore, we repeated the study by Schultz and Helmstetter and additionally measured conditioning of the startle response, which seems to be less sensitive to declarative knowledge than SCR. While we solely observed SCR conditioning in participants who reported awareness of the contingencies (*n* = 16) and not in the unaware participants (*n* = 18), we observed startle conditioning irrespective of awareness. We conclude that SCR but not startle conditioning depends on conscious discriminative fear learning.

## Introduction

There is a long-standing debate whether fear learning can occur without awareness of the relationship between the conditioned stimulus (CS) and the unconditioned stimulus (US). Some studies showed that conditioned skin conductance response (SCR) can take place in absence of contingency awareness (Esteves et al., 1994; Bechara et al., 1995; Knight et al., 2003, 2006). However, other studies showed that SCR conditioning can only be observed in parallel with the conscious expectancy of the CS to be followed by the US (Dawson and Biferno, 1973; Dawson and Furedy, 1976; Hamm and Vaitl, 1996; Purkis and Lipp, 2001; Lovibond and Shanks, 2002; Hamm and Weike, 2005; Weike et al., 2007). Importantly, under conditions that prevent both awareness and conditioning of SCR, conditioned startle potentiation could still be observed (Hamm and Vaitl, 1996; Weike et al., 2005, 2007).

The startle fear response is an automatic defensive reflex. It is potentiated in response to a CS that is associated with a US of negative valence (electrical stimulation), and can typically not be observed with USs of neutral valence (vibrotactile stimulation, reaction time task) (Lipp et al., 1994; Hamm and Vaitl, 1996). In contrast, affective valence of the US does not modify SCR conditioning, since it can occur irrespective of the valence of the US (Lipp et al., 1994; Hamm and Vaitl, 1996). As such, SCR is considered a non-specific measure of anticipatory arousal. In addition to the studies that show that SCR conditioning requires contingency learning (Dawson and Biferno, 1973; Dawson and Furedy, 1976; Hamm and Vaitl, 1996; Purkis and Lipp, 2001; Lovibond and Shanks, 2002; Hamm and Weike, 2005; Weike et al., 2005, 2007), both pharmacological and cognitive manipulations revealed that SCR is strongly related to expectancy learning. That is, propranolol administered before or after fear memory reactivation left both expectancy and SCR responding intact but reduced the startle response (Soeter and Kindt, 2010, 2011; Sevenster et al., 2012a). Moreover, the simple instruction that the CS would no longer be followed by the US eliminated both differential US-expectancy ratings and SCR (Hugdahl and Öhman, 1977; Hugdahl, 1978; Lipp and Edwards, 2002; Sevenster et al., 2012b) but not the startle fear response (Sevenster et al., 2012b). These results suggest that SCR responding appears to mirror expectancy beliefs (see also Lovibond, 2004), whereas the startle response can act independent from cognitive knowledge. In sum, there is conflicting evidence on the relation between contingency learning and SCR. One line of evidence shows that SCR does not require expectancy learning (Esteves et al., 1994; Bechara et al., 1995; Knight et al., 2003, 2006), while other evidence shows that SCR conditioning requires conscious knowledge of the contingencies (Dawson and Biferno, 1973; Dawson and Furedy, 1976; Hamm and Vaitl, 1996; Purkis and Lipp, 2001; Lovibond and Shanks, 2002; Lovibond, 2004; Hamm and Weike, 2005; Weike et al., 2005, 2007). Most evidence that SCR dissociates from expectancy learning comes from studies using a distractor task or subliminal presentation of the stimuli. These tasks often rely on post-conditioning questionnaires and can therefore not decisively demonstrate that contingency awareness was actually absent during the conditioning session (Dawson and Schell, 1985).

**Figure 1:**
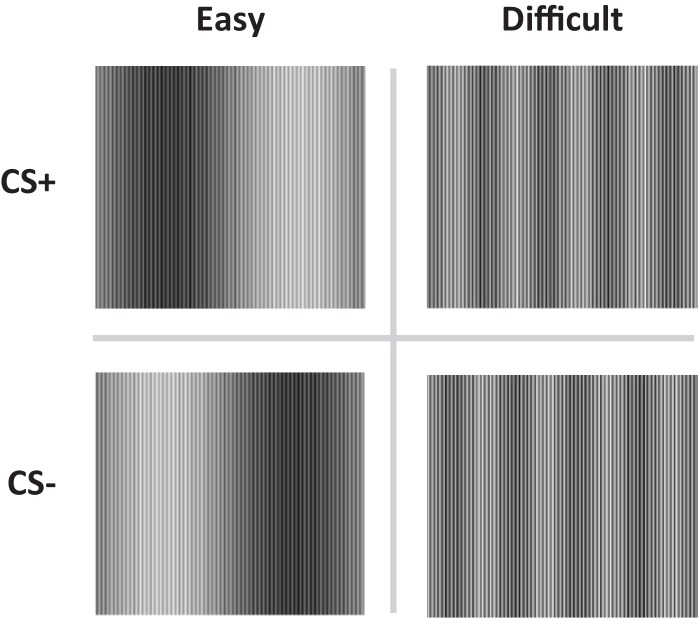
**The conditioned stimuli (CS+ and CS−) were either easy (Easy condition) or difficult to discriminate (Difficult condition)**.

Recent findings (Schultz and Helmstetter, 2010) are in line with previous studies showing that SCR does not depend on expectancy learning and challenged the observed correspondence between contingency learning and SCR responding in other labs (Hamm and Vaitl, 1996; Lovibond, 2003; Weike et al., 2005, 2007; Soeter and Kindt, 2010; Sevenster et al., 2012b, 2013). Schultz and Helmstetter (2010) manipulated contingency awareness by constructing pairs of stimuli (CS+ and CS−) that were either *easy* or *difficult* to discriminate (see Figure 1). The easy condition involved a traditional fear discrimination procedure in which the participants were aware of the contingencies, whereas in the difficult condition the participants were supposedly unaware of the contingencies. US-expectancy and SCR were registered concurrently during the conditioning session. Differential US-expectancy ratings were observed in the easy but not in the difficult condition, whereas differential SCR was observed in both the easy and difficult discrimination condition. These findings indeed suggest that SCR conditioning does not depend on contingency awareness.

The dissociation between expectancy learning and SCR conditioning observed by Schultz and Helmstetter (2010) is remarkable in the light of the typical strong convergence between expectancy learning and SCR conditioning. One explanation may be that the manipulations employed in those previous studies are simply not appropriate for dissociating the two conditioned response systems. Alternatively, studies that do not find SCR conditioning among unaware participants often use post-conditioning questionnaires (Hamm and Vaitl, 1996; Weike et al., 2005, 2007). Retrospective ratings of awareness are susceptible to forgetting or interference and may be insensitive to subtle discrimination of the CSs (Knight et al., 2003; Smith et al., 2005). The concurrent measurements of US-expectancy ratings and psychophysiological conditioned responding—a strong feature of the study by Schultz and Helmstetter (2010)—clearly overcomes this limitation. However, a shortcoming of the study by Schultz and Helmstetter (2010) is that more than half of the participants who were meant to be unaware of the learned association (difficult discrimination condition) actually demonstrated a certain degree of awareness of the CS-US contingencies. That is, a more tolerant criterion for contingency awareness revealed that more than half of the remaining, supposedly unaware participants (6 out of 10) did achieve some level of contingency knowledge in the difficult discrimination condition. The follow-up analyses showed that the six aware and the four unaware participants did not differ in SCR conditioning; it was therefore concluded that the results were not confounded by the participants’ contingency awareness. However, the small sample sizes (*n* = 4 vs. *n* = 6) make the observation of a significant difference between those two groups unlikely.

To further investigate the role of awareness in conditioned responding, we repeated the study by Schultz and Helmstetter (2010), but we increased sample sizes to allow investigation of differential SCR in participants who unintentionally acquired a certain degree of awareness and participants who were strictly unaware within the difficult condition. We expected differential SCR in the easy condition and for the difficult condition only in the participants who became aware of the contingencies during fear conditioning. Given the previously observed dissociation between US-expectancy learning and the fear potentiated startle response, we additionally tested whether conditioning of the startle response would occur irrespective of contingency awareness. A recent replication study demonstrated that trial sequence might account for the apparently unaware SCR conditioning (Singh et al., 2013). Singh et al. (2013) tested the effect of trial sequence because in previous research unaware conditioning effects could be explained by predictable trial sequence (Wiens et al., 2003). They observed that despite being classified as being unaware of the contingencies, participants in the difficult condition reported greater shock expectancy and SCR to CS+ compared to CS− on alternating trials. This effect was significantly reversed (greater responding to CS− than CS+) on non-alternating trials. Given that trial sequence effects can thus provide an alternative explanation for supposedly unaware conditioning effects, we additionally explored the effect of predictable stimulus presentation on SCR and startle conditioning.

## Materials and methods

### Participants

Thirty-seven (20 male; 17 female) healthy undergraduate students participated in the study, ranging in age between 18 and 27 years (*M* = 21.73, SD = 2.31). Participants received either partial course credit or a small amount of money for their participation. All participants gave informed consent and were notified that they could withdraw from participation at any time. Participants were medically screened to assure they were free from a physical (i.e., heart disease or epilepsy) or psychiatric disorder. The Ethics Committee of the University of Amsterdam approved the study. Participants (*n* = 37) were randomly assigned to either the easy (*n* = 18) or the difficult condition (*n* = 19). Two unaware participants in the easy condition and one aware participant in the difficult condition were excluded from analysis, resulting in *n* = 16 in the easy and *n* = 18 participants in the difficult condition.

### Apparatus

#### Stimuli

The conditioned stimuli (CS) were adapted from Schultz and Helmstetter (2010) and consisted of two pairs of complex sine wave gratings, composed of high and low frequency components (Figure 1). The high frequency component was equal for all stimuli. The low frequency components were adjusted so as to create conditions in which the two stimuli would be easy (easy condition) or difficult (difficult condition) to discriminate. Electrical stimulation of 2 ms was delivered through a pair of Ag electrodes of 20 by 25 mm with a fixed inter-electrode mid-distance of 45 mm, attached to the wrist, not the ankle (Schultz and Helmstetter, 2010). Shock deliverance was controlled by a Digitimer DS7A constant current stimulator (Hertfordshire, UK). Between the electrodes and the skin a conductive gel (Signa, Parker) was applied. US-intensity level was determined by gradually increasing shock intensity (starting at 1 mA) until subjects indicated the shock to be “uncomfortable though not painful”. Note that in the procedure used by Schultz and Helmstetter (2010) 7.5 mA was the maximum possible output current. Mean US-intensity is therefore likely to be higher in the current study. However, US-duration was substantially shorter in our study (2 vs. 500 ms), decreasing the impact of the US.

#### Fear potentiated startle

Startle response was measured through electromyography (EMG) of the right orbicularis oculi muscle. Two 6 mm sintered Ag/AgCl electrodes filled with a conductive gel (Signa, Parker) were positioned approximately 1 cm under the pupil and 1 cm below the lateral canthus, respectively; a ground electrode was placed on the forehead, 1 cm below hairline (Blumenthal et al., 2005). The startle probe was a 40 ms duration noise burst (104 dB) with a rise/fall time shorter than 1 ms, which was presented binaurally through headphones (Sennheiser, model HD 25-1 II). The EMG signal was amplified in two stages. The input stage had an input resistance of 10 MOhm, a frequency response of DC-1500 Hz and an amplification factor of 200. A 50-Hz notch filter was used to reduce interference of the mains noise. The second stage amplified the signal with a variable amplification factor of 0–100 x. The raw EMG signal was sampled at 1000 Hz and band-pass filtered (28–500 Hz, Butterworth, 4th order; Blumenthal et al., 2005) to obtain the cleanest possible data without affecting response amplitude. After rectifying and contour following (time constant = 10 ms) the peak amplitude was found by analyzing the first derivative of the resulting signal in a 30–150 ms interval following probe onset.

#### Skin conductance response

Electrodermal activity was measured using an input device with a sine-shaped excitation voltage (1 V peak-peak) of 50 Hz, which was derived from the mains frequency. Two Ag/AgCl electrodes of 20 by 16 mm were attached with adhesive tape to the medial phalanges of the first and third fingers of the non-preferred hand. The skin conductance level (SCL) signal from the input device was converted to 0.2 V/uS by a current to voltage converter. Startle response and electrodermal activity were recorded with the software program VSSRP98 at 1000 Hz. SCL was determined at 0.5 s intervals, both in the −2–0 s baseline window and the 0–7 s window after CS onset. Scores for the entire-interval response (EIR) were calculated by subtracting the mean SCL for the 2 s baseline immediately preceding CS onset from the highest SCL value recorded during the 0–7 s window after CS onset (Pineles et al., 2009), before the onset of the startle probe. This is a well-established approach of examining electrodermal reactivity (SCR) and has been used extensively in human psychophysiological research (Orr and Lanzetta, 1980; Pitman and Orr, 1986; Orr et al., 2000; Milad et al., 2005; Pineles et al., 2009; Raes et al., 2011).

#### Online unconditioned stimulus (US)-expectancy ratings

US-expectancy was measured continuously (5 samples/s), on an 11-point scale ranging from “certainly no electric stimulus” (−5) through “uncertain” (0) to “certainly an electric stimulus” (5). The scale was placed at the bottom of the screen. Participants rated US-expectancy levels by shifting the cursor on the scale with use of the mouse. Subjects were not informed about CS-US contingencies and were instructed to update their US-expectancy throughout the experiment. Continuous US-expectancy ratings during the last 4 s of CS presentation were averaged for each CS presentation. Ratings were converted to a 0–100 scale to be consistent with Schultz and Helmstetter (2010).

#### Subjective assessments

Evaluation of the US was assessed on an 11-point scale ranging from “unpleasant” (−5) to “pleasant” (5). General level of anxiety was measured with the Trait Anxiety Inventory (STAI-T; Spielberger et al., 1970) to control for**** general level of anxiety.

### Procedure

After giving informed consent participants were seated in front of a computer screen in a sound-attenuated room. The EMG, SCR and shock electrodes were attached and US-intensity level was determined by gradually increasing shock intensity (starting at 1 mA) until subjects indicated the shock to be “uncomfortable though not painful”. The experiment started with 10 startle habituation trials to stabilize baseline startle reactivity. To assess baseline startle responding during the experimental phase, startle probes alone (Noise Alone; NA) were presented in addition to the CS presentations. Throughout the entire conditioning phase participants continuously rated their US-expectancy.

#### Fear conditioning

The testing procedure was adapted from Schultz and Helmstetter (2010). In the current study NA trials were presented in addition to the CS presentations. Stimuli (CS+, CS−, NA) were presented randomly in a block of three trials. The CSs consisted of two different images depicting complex sine wave gratings. Both CSs were presented eight times, with a duration of 8 s. The experiment consisted of eight consecutive blocks. One of the images (CS+) was paired with a mild shock to the wrist (US) on all the eight trials, whereas the other picture was never paired with a shock (CS−). A startle probe (40 ms; 104 dB) was delivered 7 s after CS onset, followed by the US after another 500 ms. The US consisted of an electrical stimulus (2 ms). Note that delivery of neither the startle probe nor the US interfered with measurement of SCR as maximum SCR score was determined during 7 s following stimulus onset before the startle probe and the US onset. Intertrial intervals (ITI) varied from 15 s to 25 s with an average of 20 s. The stimuli that were easy to discriminate served as CSs in the easy condition and the stimuli that were difficult to discriminate served as CSs in the difficult condition (Figure 1). Participants did not receive information about the CS-US relationship. They were instructed to continuously place the cursor of the mouse on the position on the scale corresponding to their US-expectancy, ranging from “certainly no electric stimulus” (−5) through “uncertain” (0) to “certainly an electric stimulus” (5). After conclusion of the experimental phase, participants filled in the trait anxiety (STAI-T) questionnaire and rated US-pleasantness.

### Awareness

Continuous US-expectancy ratings during the last 4 s of CS duration were averaged for each CS presentation. Awareness was defined according to the two criteria set by Schultz and Helmstetter (2010). First, participants were classified as aware when in a sliding window of five consecutive CS presentations ratings to the CS+ were higher than 75 and ratings to the CS− were lower than 25 with at least two trials of each CS type. In the second series of analyses the effect of trial sequence on conditioned responding in the easy and difficult condition was taken into account. Second, participants were classified as aware according to the more tolerant criterion for contingency awareness when in a sliding window of four consecutive CS presentations ratings to the CS+ were higher than 50 and ratings to the CS− were lower than 50 with at least two trials of each CS type.

### Data analysis

US-intensity, US-evaluation and STAI-T scores were subjected to ANOVAs with condition (easy vs. difficult) as between-subjects factor. Startle and skin conductance response outliers were defined by means of within-participants *Z*-scores (*Z* > 3) and replaced by linear trend at point. SCRs were mean corrected, to equalize the opportunity for each subject to contribute to the group mean (Lovibond et al., 1988). The mean value used for correction was based on the eight conditioning trials. US-expectancy, skin conductance and startle potentiation data were averaged over all eight conditioning trials, resulting in a single mean response to the CS+ and a single mean response to the CS−. US-expectancy ratings, startle responses and electrodermal activity were then subjected to a mixed analysis of variance for repeated measures (ANOVA) with condition (easy vs. difficult) as between-subjects factor and stimulus (CS+ vs. CS−) and trial sequence (alternating vs. non-alternating) as within-subjects factor. The alpha level was set at 0.05 for statistical analyses.

## Results

### Easy vs. difficult

First, we analyzed the data according to Schultz and Helmstetter (2010). Awareness was defined as five consecutive CS presentations during which US-expectancy ratings to the CS+ were higher than 75 and US-expectancy ratings to the CS− were lower than 25. Based on this criterion two unaware participants in the easy condition and one aware participant in the difficult condition were excluded from further analysis, resulting in *n* = 16 in the easy and *n* = 18 participants in the difficult condition. The individually set shock intensity ranged from 6 to 40 mA (*M* = 19.09, SD = 8.83). There was no difference in US-intensity, US-evaluation and reported trait anxiety (*F*s < 1) between the aware and unaware participants (Table 1).

**Table 1:** **Mean values (SD) of the US-intensity, US-evaluation, and trait anxiety (STAI-T) for the Easy (*n* = 16) and Difficult conditions (*n* = 18)**.

	**Easy**	**Difficult**
US-intensity (mA)	21.6 (9.5)	16.8 (7.8)
US-evaluation day 1	−2.4 (1.4)	−2.6 (1.0)
Trait anxiety	34.5 (9.0)	33.8 (5.4)

#### Unconditioned stimulus (US)-expectancy ratings

**Figure 2:**
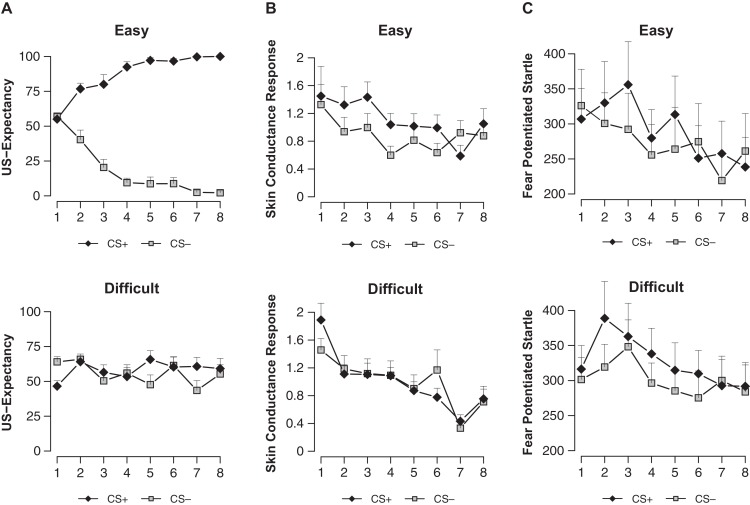
US-expectancy ratings **(A)**, skin conductance responses **(B)** and startle responses **(C)** to the CS+ and CS− for the Easy (*n* = 16) and Difficult (*n* = 18) conditions. Error bars represent s.e.m.

Analysis revealed a difference between conditions in differential US-expectancy (stimulus x condition; *F*_(1, 32)_ = 173.43, *p* < 0.001, $\eta_{p}^{2}=0.84$). US-expectancy ratings to the CS+ were higher compared to the CS− in the easy condition (main effect stimulus; *F*_(1, 15)_ = 311.99, *p* < 0.001, $\eta_{p}^{2}=0.95$), while this difference was absent in the difficult condition (main effect stimulus; *F*_(1, 17)_ < 1) (Figure 2A).

#### Skin conductance response

We observed a near-significant difference in SCR conditioning between the easy and difficult condition (stimulus × condition; *F*_(1, 32)_ = 2.99, *p* < 0.094, $\eta_{p}^{2}=0.09$) (Figure 2B). In line with the US-expectancies, we found higher SCR to the CS+ compared to the CS− in the easy condition (main effect stimulus; *F*_(1, 15)_ = 5.55, *p* < 0.032, $\eta_{p}^{2}=0.27$), while we did not observe such differential responding in the difficult condition (main effect stimulus; *F*_(1, 17)_ < 1). Thus in contrast to Schultz and Helmstetter (2010), we found that contingency awareness is a prerequisite for SCR conditioning.

#### Startle fear response

The easy and difficult condition differed neither on startle habituation (trials 1–10; stimulus × condition; *F*_(4.78, 152.99)_ < 1) nor on startle responding during the ITI (trials 1–8; stimulus × condition; *F*_(5.03, 161)_ < 1.12). Even though the easy and difficult condition did not differ in conditioning of differential startle fear response (stimulus × condition; *F*_(1, 32)_ < 1), we observed general startle conditioning evidenced by stronger startle potentiation to the CS+ compared to the CS− (main effect stimulus; *F*_(1, 32)_ = 5.84, *p* < 0.022, $\eta_{p}^{2}=0.15$) (Figure 2C). Follow-up analyses showed that conditioning of the startle was present in the difficult condition (main effect stimulus; *F*_(1, 17)_ = 4.78, *p* < 0.043, $\eta_{p}^{2}=0.22$) but not in the easy condition (main effect stimulus; *F*_(1, 15)_ < 1.66). Note that while startle conditioning was absent on the first trial (main effect stimulus; *F*_(1, 15)_ < 1), there was a significant effect of differential responding during the second to the fifth trial of early conditioning in the easy condition (trials 2–5; main effect stimulus; *F*_(1, 15)_ = 4.61, *p* < 0.05, $\eta_{p}^{2}=0.24$). This effect collapsed towards the end of conditioning (trials 6–8; main effect stimulus; *F*_(1, 15)_ < 1).

### Tolerant criterion of awareness

Schultz and Helmstetter (2010) adjusted the contingency criterion to divide the difficult condition into participants who approached the criteria for awareness and participants who did not approach awareness. They argued that by this means it could be ensured that differential SCR in the difficult condition was not influenced by a few participants who may have achieved some level of contingency knowledge. Participants were classified as approaching contingency knowledge when they rated the CS+ over 50 and the CS− under 50 on four consecutive trials in a sliding window. We applied a similar criterion to the difficult condition in the current study which resulted in a small sample (*n* = 4 unaware). Note that we did not further analyze within the difficult group. First, in contrast to Schultz and Helmstetter (2010) we do not need to exclude that differential SCR for the difficult condition was influenced by a few participants, given that differential SCR was absent in the difficult condition. Second, as mentioned in the introduction, comparing groups of *n* = 4 with *n* = 15 makes it unlikely to find any group differences.

### Easy vs. difficult: trial sequence

A recent study showed that stimulus presentation sequence could account for unaware differential SCR (Singh et al., 2013). Thus, what initially appeared to be unaware differential SCR conditioning could also be explained by expectancy “learning”, arising from a predictable trial sequence. In the current study we presented the stimuli (CS+, CS−, NA) randomly within a block, for eight consecutive blocks. Similar to the Schultz and Helmstetter study, this presentation scheme increases the probability of shock deliverance when a preceding trial is not reinforced. In contrast, when a preceding trial is reinforced the probability of shock deliverance decreases. Therefore, we re-analyzed the data with trial sequence (alternating vs. non-alternating) as an additional within subjects factor. Following Singh et al. (2013) we separately averaged conditioned responses (US-expectancy, SCR, startle response) for CS+ and CS− trials that were preceded by an opposite CS type (e.g., CS− followed by CS+; alternating trial) or a similar CS type (e.g., CS+ followed by CS+; non-alternating trial). The first two trials were not included in the analyses, since expectancies based on trial sequence could not yet have been formed for those trials (Singh et al., 2013). Note that trial sequences were not fixed, since stimulus presentation (CS+, CS−, NA) was randomized within a block of 3 trials, resulting in minimally 3 and maximally 7 alternating trials and minimally 0 and maximally 4 non-alternating trials.

#### Unconditioned stimulus (US)-expectancy ratings

**Figure 3:**
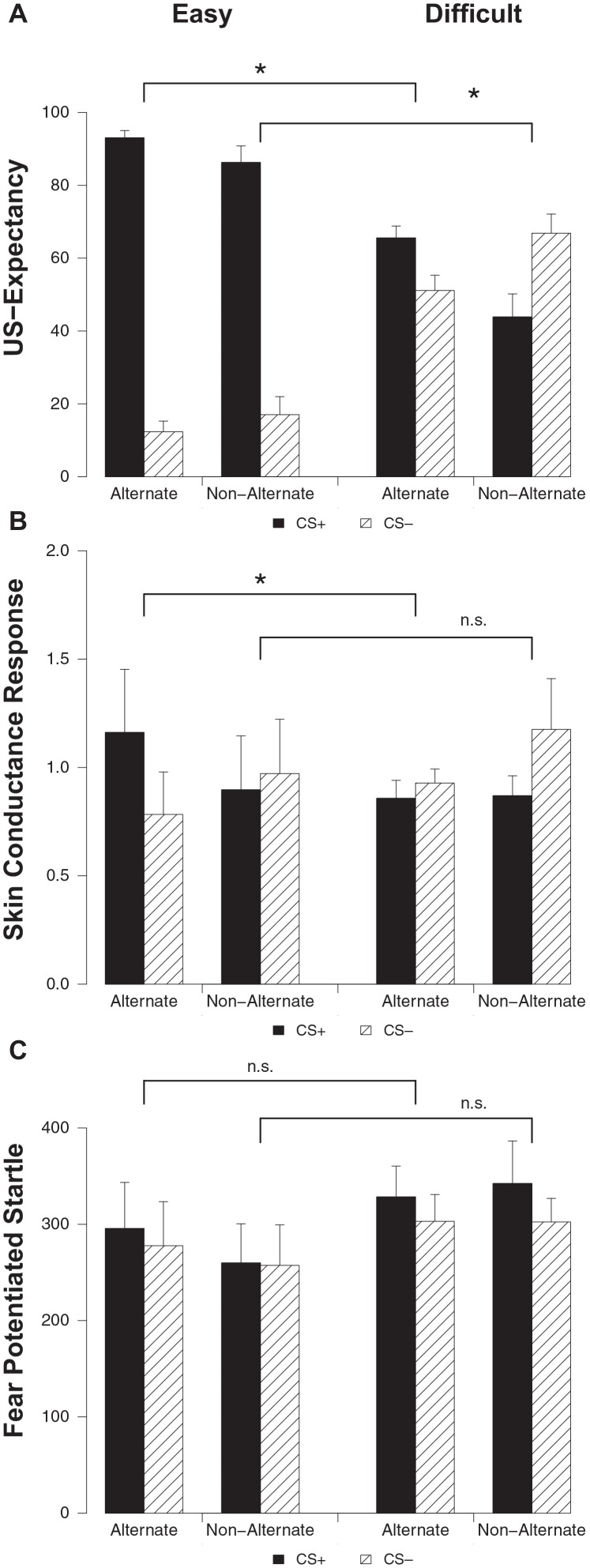
US-expectancy ratings **(A)**, skin conductance responses **(B)** and startle responses **(C)** to the CS+ and CS− on alternating and non-alternating trials for the Easy (*n* = 16) and Difficult (*n* = 18) conditions. Error bars represent s.e.m. * *p* < 0.05.

Differential ratings were greater in the easy condition compared to the difficult condition on both alternating (stimulus × condition; *F*_(1, 32)_ = 83.82, *p* < 0.001, $\eta_{p}^{2}=0.72$) and non-alternating trials (stimulus × condition; *F*_(1, 28)_ = 54.36, *p* < 0.001, $\eta_{p}^{2}=0.66$) (Figure 3A). Both on alternating (main effect stimulus; *F*_(1, 15)_ = 350.20, *p* < 0.001, $\eta_{p}^{2}=0.96$) and non-alternating trials (main effect stimulus; *F*_(1, 12)_ = 78.71, *p* < 0.001, $\eta_{p}^{2}=0.87$) responding to the CS+ was higher compared to the CS− in the easy condition. Participants in the difficult condition capitalized on trial sequence, evidenced by higher ratings to the CS+ compared to the CS− on alternating trials (main effect stimulus; *F*_(1, 17)_ = 6.56, *p* < 0.020, $\eta_{p}^{2}=0.28$), while on non-alternating trials this pattern was reversed with higher responding to the CS− compared to the CS+ (main effect stimulus; *F*_(1, 16)_ = 6.06, *p* < 0.026, $\eta_{p}^{2}=0.28$). Thus, in line with previous findings (Singh et al., 2013), we found that trial sequence can result in US-expectancy learning when stimuli are in fact difficult to discriminate.

#### Skin conductance response

Differential responding was greater in the easy condition compared to the difficult condition on the alternating trials (stimulus × condition; *F*_(1, 32)_ = 7.38, *p* < 0.011, $\eta_{p}^{2}=0.19$) (Figure 3B). Indeed, responding to the CS+ was higher compared to the CS− in the easy condition on alternating trials (main effect stimulus; *F*_(1, 15)_ = 11.00, *p* < 0.001, $\eta_{p}^{2}=0.42$). Contrary to the previous findings (Singh et al., 2013), this effect was not observed in the difficult condition (main effect stimulus; *F*_(1, 17)_ < 1). The easy and difficult condition did not differ on SCR on non-alternating trials (stimulus × condition; *F*_(1, 28)_ < 1). Follow-up analyses confirmed that differential SCR was absent in both conditions on non-alternating trials (main effect stimulus; *F*_s_ < 1.05).

#### Startle fear response

The easy and difficult condition did not differ on startle response on both alternating and non-alternating trials (stimulus × condition; *F*_s_ < 1) (Figure 3C). There was near-significantly more startle responding to the CS+ compared to the CS− on both alternating (main effect stimulus; *F*_(1, 32)_ = 3.50, *p* < 0.071, $\eta_{p}^{2}=0.10$) and non-alternating trials (main effect stimulus; *F*_(1, 28)_ = 2.96, *p* < 0.096, $\eta_{p}^{2}=0.10$). In sum, we replicated the US-expectancy results of Singh et al. (2013). Predictable trial sequence did facilitate expectancy learning in spite of difficult to discriminate stimuli. However, trial sequence only facilitated differential SCR in the easy but not in the difficult condition. Differential startle responding was not affected by trial sequence.

## Discussion

The aim of the present study was to investigate whether startle and SCR conditioning correspond with contingency awareness. First, the main finding was that startle conditioning can occur independent of contingency awareness. We observed differential conditioning of the fear potentiated startle, in spite of difficult to discriminate CS. While the easy and difficult conditions did not differ in startle responding, we unexpectedly did not observe startle conditioning in the easy condition. Thus, startle responding to the CS+ was not higher than startle responding to the CS− when analyzing the eight conditioning trials together. However, participants in the easy condition showed startle conditioning at the beginning of the fear acquisition phase, but this effect was no longer present by the end of conditioning. Visual inspection of the graphs suggests a similar pattern of conditioning over trials for the startle response in the difficult condition and SCR in the easy condition. The observation of a transient physiological conditioning effect contrasts with our previous studies in which we did observe SCR and startle conditioning during late acquisition (Kindt et al., 2009; Soeter and Kindt, 2010; Sevenster et al., 2012a). In these studies we used fear-relevant stimuli, while in the current study CSs consisted of neutral pictures. The absence of differential psychophysiological responding at the end of conditioning in the current study might be explained by faster habituation to neutral CSs than to fear-relevant stimuli (Lovibond et al., 1994).

Second, SCR appeared to covary with the US-expectancy ratings, demonstrated by differential SCR conditioning in the easy but not in the difficult condition. While Schultz and Helmstetter (2010) found more electrodermal responding to the CS+ compared to the CS−, irrespective of the difficulty to perceptually discriminate these stimuli, we did not replicate these findings. More than half of the participants in the difficult condition showed contingency awareness according to the tolerant criterion in their study, but only 4 out of the 19 participants in the difficult condition met the same awareness criterion in the current study. Therefore, SCR conditioning in the study by Schultz and Helmstetter (2010) can arguably be attributed to the relatively large sample of participants who unintentionally acquired some level of contingency awareness in the difficult condition. It is hard to explain the differences in proportion of aware vs. unaware subjects between the study by Schultz and Helmstetter and the current study. We might therefore question the reliability of this approach to assess contingency awareness. Note, however, that the validity of the currently used approach is higher relative to the use of post-experimental questionnaires, as the online measurement of expectancy learning overcomes many of the limitations of retrospective contingency assessment (see Lovibond and Shanks, 2002; Schultz and Helmstetter, 2010).

A previously proposed alternative explanation for the findings by Schultz and Helmstetter (2010) is that participants classified as aware in the difficult condition could have capitalized on the trial sequence to predict the occurrence of the US, as was convincingly argued in a recent replication study (Singh et al., 2013). When presentation of the CS+ and CS− followed an alternating sequence, participants not only showed higher US-expectancy to the CS+ compared to the CS−, but also differential SCR. Thus, although incapable of discriminating the two CSs, predictable trial sequence may have resulted in unintended “contingency awareness” and subsequent differential SCR conditioning. We partly replicated this effect, by showing that expectancy “learning” occurred on alternating but not on non-alternating trials when stimuli were difficult to discriminate. If unintended contingency awareness indeed results in SCR conditioning, as demonstrated by Singh et al. (2013), SCR conditioning should have been present on alternating trials, irrespective of condition. However, while we observed differential SCR on alternating trials in the easy condition, expectancy “learning” did not result in differential SCR in the difficult condition. In the current study participants in the difficult condition did apparently not benefit from a predictable trial sequence. Our design differed from Singh et al. (2013) in that their conditioning procedure was extended with two more blocks of CS presentations. These additional trials might have been essential for the participants to capitalize on rules about the trial sequence. Surprisingly, we found that differential SCR was affected by trial sequence in aware participants. This shows that awareness may be required but not necessarily sufficient for differential SCR to occur, since SCR was absent for the non-alternating trials in participants who reported awareness of the contingency. This stresses the urge to control for trial sequence in fear conditioning research, especially when using SCR as a measure of conditioned responding. Notably, differential startle responding occurred irrespective of condition and trial sequence.

Fear is considered to be characterized by both high arousal and negative valence (Lang, 1995). While it would be tempting to speculate on the differential role of valence and arousal in SCR and startle conditioning (Bradley and Vrana, 1993; Bradley et al., 1993; Lang, 1995), it is hard to actually dissociate valence and arousal by means of these psychophysiological measures. Potentiation of the startle reflex is generally associated with valence and SCR with arousal. But the startle reflex is also modulated by arousal: negative pictures evaluated as most arousing produce greatest startle potentiation, while positive pictures evaluated as most arousing produce greatest startle inhibition. In contrast, an increase of SCR has been demonstrated for both negative and positive pictures which are evaluated as most arousing (Cuthbert et al., 1996). Thus, negative valence engages the defensive system and arousal in turn modulates the degree of activation of the defensive system. In sum, it is hard to distinguish between the influence of valence and arousal on startle conditioning under circumstances that induce both as is probably the case in fear conditioning. Nevertheless, given that valence is a necessary condition for startle potentiation to occur while SCR can be established independent from negative valence, the startle response does seem a more specific index of fear than SCR.

In line with previous evidence, the current study shows that the startle response does not mirror expectancy learning and SCR. The dissociation between the response systems raises important questions concerning the function of unaware startle conditioning. Remarkably, the unaware startle conditioning effect seems to be of a transient nature, since it is no longer observed during an extinction phase immediately following conditioning (Weike et al., 2007). Imaging research already showed that conditioning-related neural responses can take place in absence of awareness and SCR conditioning (Tabbert et al., 2011). It would be interesting to see whether evidence for an intact memory representation of unaware conditioning can still be observed at a later retention test. If there is no indication that the immediately developed unaware conditioned responding outlasts the conditioning phase—either behaviorally or neurally—the question is whether there is any adaptive value in the online but transient development of automatic conditioned responding.

The current findings are not easily reconciled with a single-process model of fear learning. This account would predict that contingency awareness affects the startle response to a similar degree as expectancy learning and SCR. Yet, we demonstrated that, contrary to the startle response, acquisition of both US-expectancy and SCR no longer takes place when using difficult-to-discriminate stimuli. As such, the current findings suggest that fear learning involves at least two processes. The US-expectancy ratings and the closely associated SCR seem to reflect the propositional level of associative fear learning, whereas the startle response may be better explained in terms of a more automatic, low-level index of fear. The finding that the conditioned responses involved in associative learning rely on different neural circuits further challenges the single-process account. That is, declarative knowledge of the CS-US contingencies relies among other brain areas on the hippocampal complex (Hamm and Weike, 2005; Weike et al., 2005; Hunsaker and Kesner, 2013). In contrast, the startle response is an amygdala-initiated response and therefore considered to reflect the brain’s subcortical defense system (Walker and Davis, 2002; LeDoux, 2003) (for a more elaborate discussion on the single vs. dual process account and its neural underpinnings see Sevenster et al., 2012b).

Note that while we consider SCR not an optimal correlate for fear, we do not argue that SCR is not a suitable measure in human fear conditioning research. During standard fear conditioning and extinction procedures, the measures corresponding to different response systems generally converge into similar learning patterns (Vansteenwegen et al., 2005; Soeter and Kindt, 2010, 2011; Vervliet et al., 2010; Sevenster et al., 2012a,b). Only specific manipulations—either pharmacological or behavioral—may reveal the differences between these conditioned responses (Soeter and Kindt, 2010, 2011; Sevenster et al., 2012a,b, Sevenster et al., 2013). In human fear conditioning research, multiple indices of the behavioral expression of fear (e.g., US expectancies, distress ratings, SCR, startle potentiation) are usually obtained for reasons of cross-validation. Given that these measures of fear learning do not necessarily converge (Hamm and Weike, 2005; Kindt et al., 2009; Soeter and Kindt, 2010; Sevenster et al., 2012a,b, Sevenster et al., 2013; Beckers et al., 2013), future research should incorporate this apparent divergence by predicting *a priori* (differential) effects for the different dependent variables used to measure fear learning.

## Author contributions

Dieuwke Sevenster, Tom Beckers and Merel Kindt designed the study. Dieuwke Sevenster analyzed the data. Dieuwke Sevenster and Merel Kindt wrote the manuscript. Tom Beckers provided revision of the manuscript.

## Conflict of interest statement

The authors declare that the research was conducted in the absence of any commercial or financial relationships that could be construed as a potential conflict of interest.
